# The Effect of Cellulose Nanocrystals on the Molecular Organization, Thermomechanical, and Shape Memory Properties of Gelatin-Matrix Composite Films

**DOI:** 10.3390/gels10120766

**Published:** 2024-11-25

**Authors:** Cristina Padilla, Marzena Pępczyńska, Cristian Vizueta, Franck Quero, Paulo Díaz-Calderón, William Macnaughtan, Tim Foster, Javier Enrione

**Affiliations:** 1 Biopolymer Research & Engineering Laboratory (BIOPREL), Escuela de Nutrición y Dietética, Facultad de Medicina, Universidad de Los Andes, Santiago 7550000, Chile; capadillar@uandes.cl (C.P.); pdiaz@uandes.cl (P.D.-C.); 2 Centro de Investigación e Innovación Biomédica (CIIB), Facultad de Medicina, Universidad de los Andes, Santiago 7550000, Chile; 3 R&D Physical Properties, Laboratorios Liconsa-CHEMO, S.A. Polígono Industrial Miralcampo, Avda. Miralcampo 7, 19200 Azuqueca de Henares, Guadalajara, Spain; mpepczynska@gmail.com; 4 IMPACT, Center of Interventional Medicine for Precision and Advanced Cellular Therapy, Universidad de los Andes, Santiago 7550000, Chile; cvizueta@uandes.cl; 5 Laboratorio de Nanocelulosa y Biomateriales, Departamento de Ingeniería Química, Biotecnología y Materiales, Facultad de Ciencias Físicas y Matemáticas, Universidad de Chile, Santiago 8370456, Chile; fquero@ing.uchile.cl; 6 Division of Food, Nutrition and Dietetics, School of Biosciences, Sutton Bonington Campus, University of Nottingham, Loughborough LE12 5RD, UK; bill.macnaughtan@nottingham.ac.uk (W.M.); tim.foster@nottingham.ac.uk (T.F.)

**Keywords:** cellulose nanocrystals, gelatin, crystallinity, molecular interactions, thermomechanical properties, shape memory

## Abstract

Gelatin is a natural hydrocolloid with excellent film-forming properties, high processability, and tremendous potential in the field of edible coatings and food packaging. However, its reinforcing by materials such as cellulose nanocrystals (CNC) is often necessary to improve its mechanical behavior, including shape memory properties. Since the interaction between these polymers is complex and its mechanism still remains unclear, this work aimed to study the effect of low concentrations of CNC (2, 6, and 10 weight%) on the molecular organization, thermomechanical, and shape memory properties in mammalian gelatin-based composite films at low moisture content (~10 weight% dry base). The results showed that the presence of CNCs (with type I and type II crystals) interfered with the formation of the gelatin triple helix, with a decrease from 21.7% crystallinity to 12% in samples with 10% CNC but increasing the overall crystallinity (from 21.7% to 22.6% in samples with 10% CNC), which produced a decrease in the water monolayer in the composites. These changes in crystallinity also impacted significantly their mechanical properties, with higher E’ values (from 1 × 10^4^ to 1.3 × 10^4^ Pa at 20 °C) and improved thermal stability at higher CNC content. Additionally, the evaluation of their shape memory properties indicated that while molecular interactions between the two components occur, CNCs negatively impacted the magnitude and kinetics of the shape recovery of the composites (more particularly at 10 weight% CNC, reducing shape recovery from 90% to 70%) by reducing the netting point associated with the lower crystallinity of the gelatin. We believe that our results contribute in elucidating the interactions of gelatin–CNC composites at various structural levels and highlights that even though CNC acts as a reinforcement material on gelatin matrices, their interaction are complex and do not imply synergism in their properties. Further investigation is, however, needed to understand CNC–gelatin interfacial interactions with the aim of modulating their interactions depending on their desired application.

## 1. Introduction

Gelatin is a natural hydrocolloid that has been widely used for many years in the food and pharmaceutical industries [1,2,3]. It is obtained from the hydrolysis of collagen, a fibrous protein present in the connective tissue of mammals, such as skin, hair, cartilage, and bones [4]. Gelatin extraction from collagen can be obtained by two main methods, namely acid or basic hydrolysis. Acid hydrolysis derives type A gelatin with an isoelectric point of 7–9. Basic hydrolysis generates type B gelatin that has an isoelectric point of 5–6 [4,5,6]. Also, it has been described that gelatin’s physical properties can be controlled by the extraction process conditions (various pH and temperatures) to potentially tune its technological properties for specific applications [7,8].

Collagen’s native structure consists of a triple helix formed by three polypeptide chains, called α-chains, mainly held together by inter-chain hydrogen bonding [9,10]. These polymeric chains consist of repetitive tri-peptide glycine-x-y sequences, with the imino acids proline (pro) and hydroxyproline (hyp) most frequently situated in the x and y positions, respectively [11,12]. This sequence plays a very important role in gelatin’s structure and is related to the formation and stability of random coil and helical structures that are associated with its partial renaturation [6,12]. In fact, it has been reported that a higher content of pro and hyp i.e., in mammalian as opposed to cold water fish gelatin, is associated with a higher triple helical content, translating into higher gelation temperatures and mechanical properties, including gel strength [13,14,15]. Gelatin can be suspended in water at ∼60 °C, where it exists in a random coil conformation above its melting temperature. Upon cooling and below their melting temperature, protein chains start to interact with each other, and gelation occurs forming a partially collagen-like triple helix structure, while a certain proportion remains in the random coil conformation [6,9,16]. The thermo-reversible coil–helix transition provides gelatin with excellent film-forming properties, high processability [2,17,18], and tremendous potential in the field of edible coatings and food packaging [19,20,21,22,23,24,25]. As in synthetic polymers, mechanical properties are important for these applications, and generating composite materials is a common strategy to optimize their mechanical behavior. Several publications report various gelatin-matrix composites containing nanomaterials such as silver nanoparticles, nanoclays [26], and montmorillonite [27]. Also, synthetic polymers such as polyvinyl alcohol (PVA) [28] have been used to modulate gelatin’s mechanical and physicochemical properties. Gelatin-matrix composites combined with natural polymers have been studied as a strategy to obtain a reinforced gelatin composite. These include chitin nanoparticles [29], chitosan [30], and nanocellulose [31,32], to name only a few. In the case of cellulose, its chains are firmly held together by hydrogen bonds, forming microfibrils and fibrils with very high tensile strength [33]. The moduli of cellulose fibrils from various sources have been determined by X-ray diffraction, atomic force microscopy (AFM), and theoretical methods, where values between 100 and 160 GPa have been reported [34,35]. Thus, cellulose fibrils are good candidate to be used as a natural, renewable, and biodegradable reinforcing material to create fully natural polymer composite materials.

Cellulose is a polysaccharide formed by a linear chain of hundreds to thousands of repetitive β-1,4 linked D-glucose units [33]. It is the most common organic polymer on earth, and it is considered an almost inexhaustible source of raw material [36]. In the last 30 years, nanocellulose, including cellulose nanofibers (CNF), have been studied as a reinforcement for composite materials due to their high crystallinity, stiffness, and tensile strength. CNF display diameters in the range of tens of nanometers and may be classified as microfibrillated cellulose (MFC) (also referred to as nanofibrillated cellulose, NFC), bacterial cellulose (BC), and cellulose nanocrystals (CNCs) with different length/diameter aspect ratios [37]. Additionally, cellulose nanospheres (CNPs) have been described in recent years as a new type of nanocellulose with spherical morphology [38]. Nanocellulose’s reinforcement ability has been demonstrated mainly with synthetic polymers [39]. For example, studies of PVA–nanocellulose composites and polylactic acid (PLA)–nanocellulose have shown mechanical reinforcement of the matrices [40,41]. In the case of mammalian gelatin, studies have focused on the use of relatively low contents of nanocellulose from bacterial or plant origins to reinforce the gelatin matrix. The obtained results, however, have provided conflicting information in terms of mechanical properties and molecular interactions. Studies of bovine gelatin with bacterial CNCs resulted in the improvement of the composites’ mechanical properties, thermal stability, and reduced moisture affinity [42]. Similar results were found by Yang et al. 2018, using gelatin–MFC composites [43]. Also, interfacial stress transfer occurring from the gelatin matrix to BC and to MFC has been demonstrated [31,32]. However, stress transfer quantified by Raman spectroscopy showed that high values of stress transfer do not necessarily correlate well with an improved mechanical performance of gelatin-based composites from different origins (mammalian or cold-water fish) [31]. Moreover, other studies suggest that MFC and CNC decrease gelatin’s tensile strength, suggesting low stress transfer between the two components, although this parameter was not measured in this case [44]. Additionally, other authors have recently demonstrated that different molecular morphologies of nanocellulose modified the structure and characteristics of gelatin films, showing that a low weight (wt.) percentage (2%) of CNF and CNC improved the films’ mechanical properties due to cellulose–gelatin hydrogen bonding interaction and a good distribution of cellulose in the matrix compared to the higher distribution of CNF (5 wt.%) and the addition of CNCs. Also, they demonstrated that among the three nanocellulose used, films with 2 wt.% CNCs incorporation showed the best performance in increasing the mechanical and barrier properties [45]. These studies showed that the interaction between these polymers is complex, and there is still a need for information to establish and develop composites with controlled properties to provide those with the widest range of potential applications. In addition, not all studies have considered or controlled tightly the moisture content present on these matrices. Due to the hygroscopic nature of gelatin, these materials are sensitive to moisture, and their properties can change with variations of relative humidity (RH) [46,47]. Water molecules interact with the components of the composite blends by hydrogen bonds affecting their mechanical, permeability, and optical properties [48]. Water can significantly alter molecular mobility, acting as a plasticizer [49]. Therefore, we believe that a characterization of these materials at low and controlled moisture content can help to improve the understanding of gelatin–CNC molecular interactions.

The work described in this manuscript studies the effect of low concentrations of CNC (2–10 wt.%) on the molecular organization and thermomechanical interactions in mammalian gelatin-based composite films at low moisture content (~10wt.% dry base, d.b). Composites formed by gelatin and various CNC contents were studied through changes in molecular and water interactions and crystallinity determinations. These results were complemented with respect to the determination of thermomechanical and shape memory properties of the composite materials. We believe that these results provide new information for elucidating the molecular interactions between gelatin and CNC where water certainly plays a crucial role. This new understanding may contribute to the development of fully natural materials with versatile and finely controlled physical properties, including thermomechanical and shape memory properties.

## 2. Results and Discussion

### 2.1. CNC Characterization

The CNCs used in this study have been previously characterized by atomic force microscopy (AFM) by our group and were dimensions of approximately 100–200 nm in length and 10–20 nm in width [50], confirming their rod-like morphology and nanometric size [34,51].

**Figure 1:**
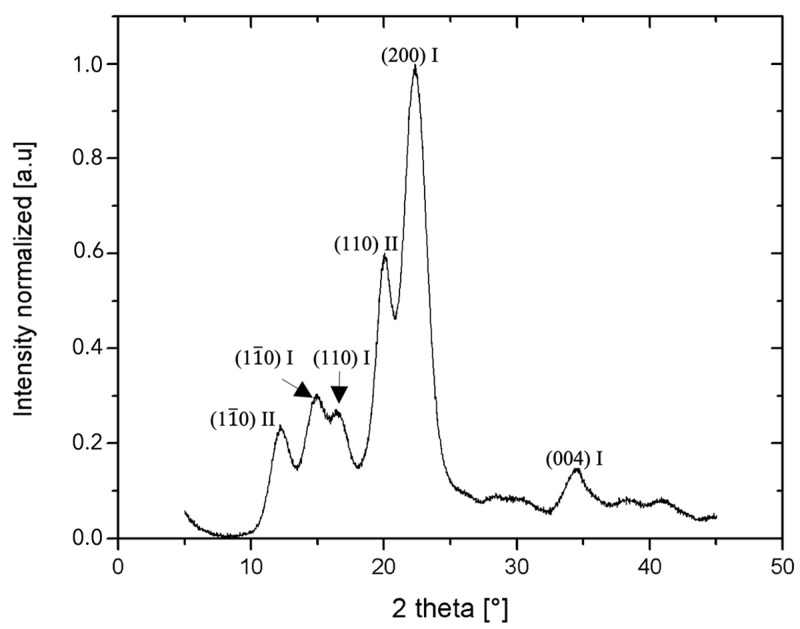
The powder X-ray diffraction pattern showing the characteristic diffraction planes of CNCs.

The powder XRD patterns of the CNCs showed the characteristic diffraction peaks of cellulose I polymorph (Figure 1), with diffraction planes of (1$\bar{1}$0), (110), (200), and (004) corresponding to the diffraction peaks located at 2θ~15°, 17°, 22.6°, and 34.5°, respectively [52,53,54]. Interestingly, CNCs also presented characteristic peaks associated with cellulose II polymorph with diffraction planes of (1$\bar{1}$0) and (110) corresponding to the diffraction peaks located at 2θ~12° and 20° [52,53,54], suggesting that CNCs have both type I and II crystals in their crystalline structure, probably due to the hydrolysis conditions used by the manufacturer [52]. The crystallinity of the CNCs was found to be relatively high with a value of ~88%, as calculated by integration method. The literature shows that by changing acid hydrolysis parameters on cotton, such as the type of acid used, crystalline polymorphs I and II that possess different molecular structures, morphology, and thermal stability can be achieved [55]. Moreover, another study produced four types of CNC with modulated morphologies by first obtaining different cellulose polymorphs from cotton and then producing CNCs through efficient hydrogen peroxide hydrolysis. These CNCs, exhibited differences in size and thermal stability [56].

### 2.2. Analysis of of Gelatin–CNC Composites

#### 2.2.1. Molecular Interactions

**Figure 2:**
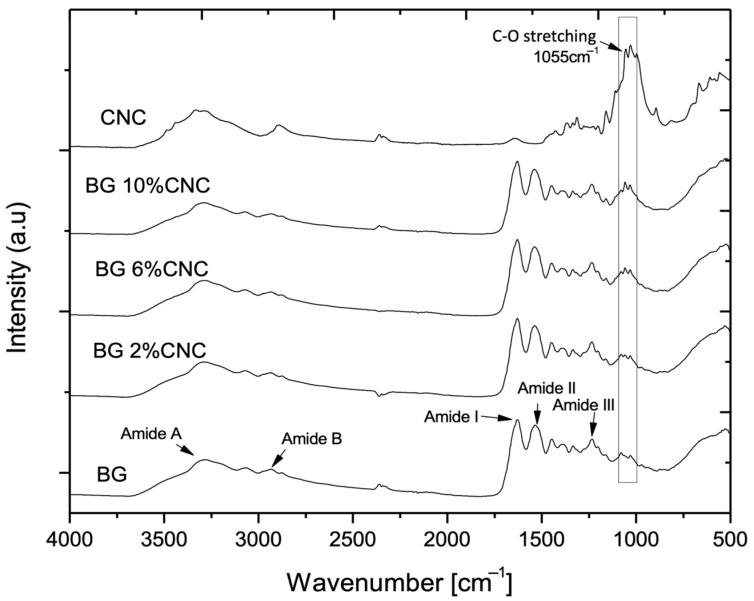
FT-IR spectra of gelatin, CNC, and gelatin–CNC composite films equilibrated to 33% RH, ~10 wt.% water content d.b. The highlighted absorption peak located at 1055 cm^−1^ is related to the vibrational motions of the C-O stretching of primary alcohols that belong to the molecular structure of cellulose.

FT-IR has been widely used to study the secondary structure of collagen and gelatin [57,58]. The spectrum obtained for gelatin showed its characteristic chemical groups, such as the absorption bands of amide A (N-H stretching) located at a wavenumber position of ~3288 cm^−1^, amide B (C-H stretching) at 2930 cm^−1^, amide I (C=O and N-H stretching) at 1632 cm^−1^, amide II (C-N and N-H stretching) at ~1530 cm^−1^, and amide III (C-N and N-H stretching) at ~1233 cm^−1^ [58,59,60,61,62] (Figure 2). Spectral changes in these chemical groups, mainly identified in the amide A, I, II, and III regions, are related to the degree of molecular order and are involved with the triple helical structure of collagen [61]. Also, changes in collagen’s secondary structure and the decrease in relative intensity of some of these signals have been attributed to collagen denaturation [58]. On the other hand, CNCs presence in the composite was detected owing to the appearance of a band located at ~1055 cm^−1^, related to C-O stretching of primary alcohols of the cellulose molecular structure (Figure 2) [42,63,64]. The increase in the relative intensity of this band is proportional to the amount of CNC present in the composites (Figure 2). The spectra of gelatin and gelatin–CNC composite films showed similar patterns; however, a slight increase in amide A signal (Table 1) related to N-H stretching suggested a decrease in hydrogen bonding through these groups and thus a decrease in triple helix content due to CNC presence in the samples [65,66]. Additionally, the slight increase in the amide I signal observed when CNC are present in the composites (Table 1) could also suggest a loss in the molecular order of gelatin chains [62].

#### 2.2.2. Surface Mapping by Raman Spectroscopy

**Figure 3:**
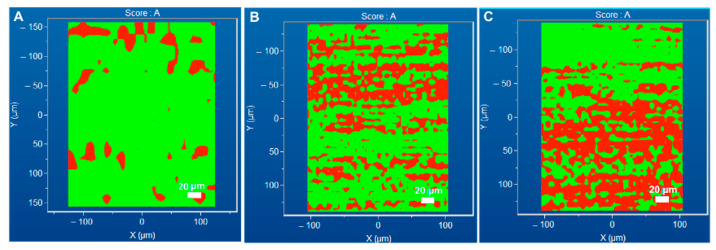
Gelatin’s and CNCs’ surface distribution at the surface of gelatin–CNC composite films equilibrated to 33% RH, ~10wt.% water content d.b. Raman images show gelatin’s (green) and CNCs’ (red) distribution at the surface of composite films. (**A**) BG 2 wt.% CNC; (**B**) BG 6 wt.% CNC; (**C**) BG 10 wt.% CNC.

The 2D chemical images of the surface of the gelatin–CNC composite films were acquired by Raman spectroscopy in mapping mode. These images, reported in Figure 3, show that the CNCs were distributed relatively homogeneously at the surface of the gelatin matrix. Indeed, an increase in CNC concentration produced an increase in the CNC signals at the films’ surface. The presence of CNC, however, seems high with respect to the concentrations used. It is important to bear in mind that this analysis aimed to highlight the distribution of both materials at the surface of the composite films rather than the quantification of the CNC concentration itself in the bulk materials [67].

#### 2.2.3. Determination of Crystallinity

**Figure 4:**
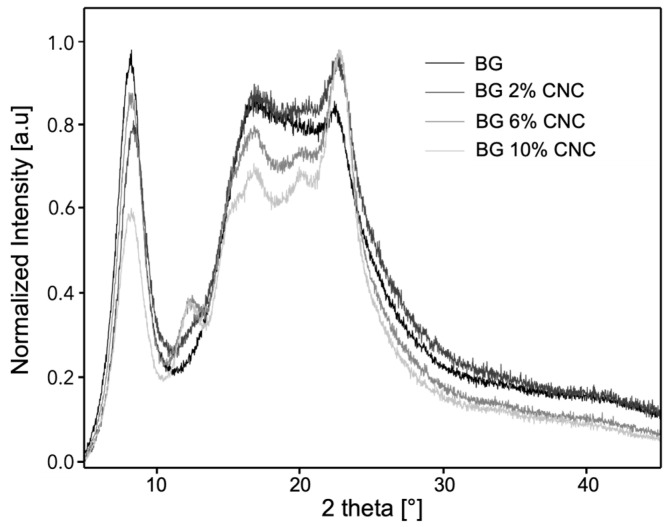
X-ray diffraction patterns of gelatin, CNC, and gelatin–CNC composite films equilibrated to 33% RH, ~10 wt.% water content d.b with various CNC weight percentages.

XRD patterns of gelatin and gelatin–CNC composite films are presented in Figure 4. In the case of gelatin, a defined and high-intensity peak can be observed at a 2θ diffraction angle position of ~7°, which has been described as the crystalline fraction of the gelatin, associated with the triple helix content [7,68,69]. The intensity of this peak can be related to the high content of gelatin’s triple helix structures, which is related to the thermo-reversibility of gelatin and the cold casting conditions used in this study. The second broad peak observed in the 2θ range of 15–25° is associated with the amorphous fraction or random coil present in the gelatin [7,68]. In the case of the gelatin–CNC composite films, diffraction peaks of both components can be identified, such as the high-intensity peak of gelatin located at 2θ~7° and the diffraction planes of (110) and (200) corresponding to 2θ ~17°and 22.6°, respectively, from cellulose I polymorph [52,53,54] and diffraction planes of (1$\bar{1}$0) and (110) corresponding to 2θ = 12° and 20° (6 and 10 wt.% CNC) from cellulose II polymorph (Figure 4).

Regarding the crystalline fraction of gelatin, one can observe that the presence of CNC reduced the relative intensity of the diffraction peak located at 2θ ~7° and gelatin’s crystallinity, suggesting a significant decrease of the triple helix for composite films with 2, 6, and more particularly 10 wt.% CNC (Table 2). However, one can observe an increase in the overall composite crystallinity % due to the presence of CNC owing to its high crystallinity (~88%), contributing significantly to the overall crystallinity of the composite films. The work of Quero et al., 2015, described the effect of bacterial (BC) cellulose on the gelatin triple helix formation determined by XRD, also showing a significant increase in the presence of the peaks related to the BC. In this study, they concluded that the presence of BC (0.5, 2, 6, and 10 wt.% in gelatin) did not affect gelatin triple helix content [32]. The difference in the reported work and this manuscript may be related to the size and morphology of the nanocellulose used. CNCs having a rod-like morphology are significantly smaller in size than BC, which has a ribbon-like morphology; hence, CNCs may have the ability to alter the process of triple helix formation upon cooling. A recent work studied the effect of CNCs on gelatin’s triple helix content by XRD, showing that for composites prepared at pH 8 (over gelatin’s IEP), low CNC contents (0.5 wt.%) increased gelatin’s crystallinity, while at higher concentrations (5 wt.%), gelatin’s crystallinity decreased [70]. Indeed, it has been reported that in the case of CNC, it can be a more evenly distributed within the gelatin matrix compared to other nanocellulose structures, such as cellulose nanofibrils or nanospheres, affecting the triple helix formation during film formation [45]. This effect has been explained by hydrogen bonding and hydrophobic interactions between gelatin and cellulose nano particles, leading to a decrease in the formation of the gelatin triple helix structure [66]. Moreover, it is well known that the production of CNC by different methods generates major changes in its molecular structure but also important effects on surface electrostatic charge. This can have a significant impact on its interaction with other electrically charged polymers given that the Z potential of CNC is generally markedly negative. This, in turn, significantly affects its interaction with polymers that may be positively charged, as is the case with gelatin, thus influencing the mobility of this polymer to form the triple helix [71].

Additionally, XRD studies of gelatin and oligosaccharides have shown that the relative intensity of the diffraction peak located at 2θ = 7° decreases in the presence of glucose, sucrose, and maltodextrin and that this effect was related to the molecular weight of the oligosaccharides. Interestingly, the reduction of the triple helix content was more significant in the presence of glucose; however, the concentrations of oligosaccharides used in this work were higher (20–60 wt.%) than the concentrations used in this study (5–10 wt.%) [72].

#### 2.2.4. Water Interactions 

**Figure 5:**
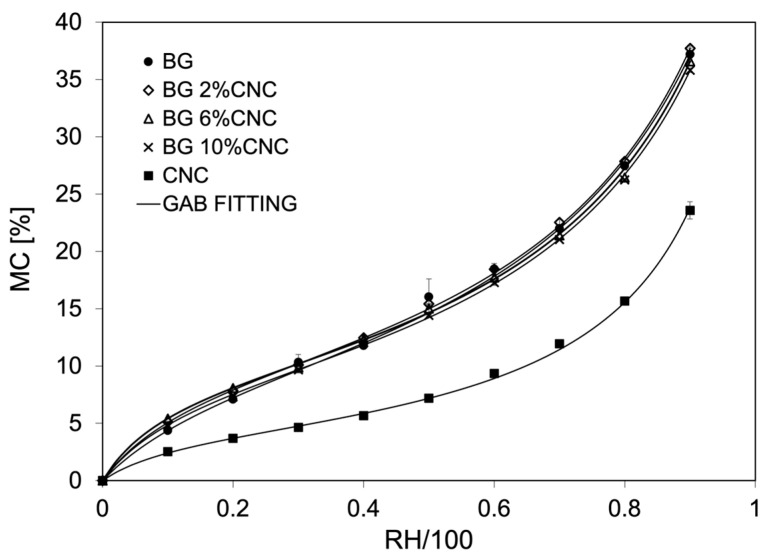
Sorption isotherms at 20 °C of gelatin, CNC, and gelatin–CNC composite films with different CNC weight fractions. Lines in the graphs represent the fitting to the experimental data using the GAB equation. The inset shows isotherms at low moisture content.

Water sorption isotherms at 20 °C were determined for gelatin, CNC, and gelatin–CNC composite films (Figure 5). Pure gelatin samples showed higher equilibrium moisture content in relation to pure CNC, probably due to the high crystallinity of CNC and therefore lower water interaction. In the case of the composites with 6 and 10 wt.% of CNC, they showed a slight decrease in equilibrium moisture content at a relative humidity higher than 40%. At this latter value, the glass transition temperature (Tg) of the gelatin present in the composite reaches ~20 °C, increasing the molecular mobility, allowing for greater interactions between the polymer chains and water molecules. The extension of this small decrease in moisture sorption was correlated with the weight fraction of CNC present in the composites.

The fitting of the experimental data using the GAB model [73] was adequate for all the samples studied, obtaining low MSE values (Table 3). The moisture content at the monolayer values (m_o_) showed a decrease, although not significant in the presence of CNC in the composites. Also, with the presence of CNC, an increase in C_GAB_ values was observed. This would imply that CNC can affect the water sorption in the composites, possibly by locating water molecules at the surface with higher sorption energy due to available hydroxyl groups in the CNC structure. However, K values were not changed, suggesting that CNC did not affect the water sorption at the multilayer and bulk water interface. These results agree with a previous study looking at gelatin–CNC from bacterial cellulose, where 2 and 4% of CNC reduced the equilibrium moisture content of gelatin above 30%RH. This was explained by the interaction of CNC with the hydrophilic sites of gelatin, displacing water–gelatin interactions [42].

#### 2.2.5. Thermal Characterization 

**Figure 6:**
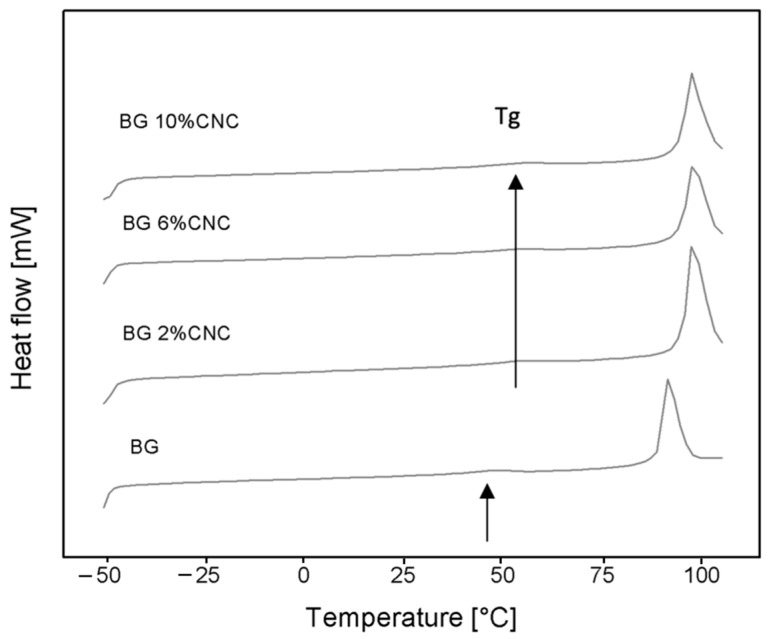
DSC thermograms (first heating scans) of gelatin and gelatin–CNC composite films with different CNC weight fractions equilibrated under 33% RH, ~10wt.% water content d.b. Black arrows indicate the glass transition temperature (Tg) shift with the presence of CNC.

Thermograms and thermal parameters of the gelatin and gelatin–CNC composite films are presented in Figure 6 and Table 4, respectively. In the case of the thermograms, the data show that T_g_ and T_m_ values of gelatin were increased when CNC was present (Figure 6). However, the ΔH_m_ was not varied in the composites compared to the gelatin. These data suggest that CNC can reduce gelatin molecular mobility in the composites, probably due to changes in overall viscosity and physical interaction between both polymers, suggesting a reinforcing effect. These results agree with a previous study that evaluated the effect of CNC and CNF in gelatin films with low moisture contents [44], reporting, for both types of composites, an increase in T_g_ associated with the gelatin when the nanocellulose was present, whereas ΔH_m_ values remained unchanged. The latter would suggest there was no difference in the triple helix content in the gelatin fraction in the composite, following different results than from XRD data. This could be explained by the fact that XRD provides information of molecular order at a higher level of molecular organization than the information provided by DSC [72].

#### 2.2.6. Mechanical Characterization 

**Figure 7:**
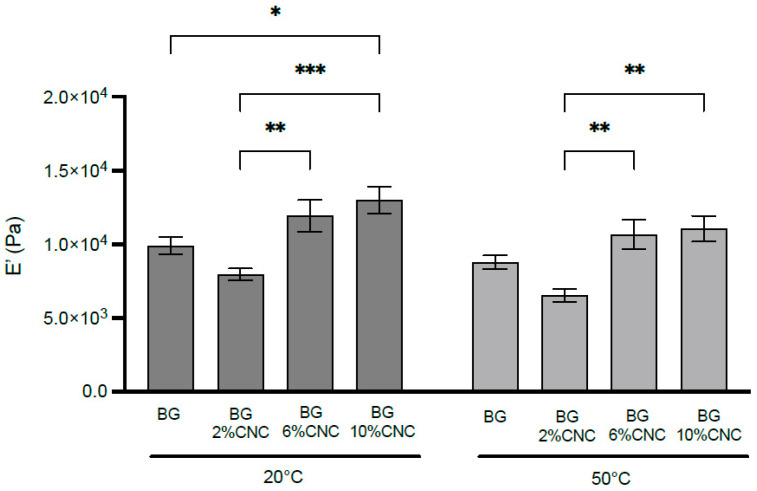
Storage modulus (E’) of gelatin and gelatin–CNC composite films with different CNC weight fractions equilibrated at RH 33% at 20 and 50 °C. Data from 4 independent experiments. * *p* < 0.05, ** *p* < 0.005, *** *p* < 0.0005.

In Figure 7, the DMA analysis evidenced differences in the elastic modulus (E’) when CNC was present in the composites equilibrated at 33% RH. At the temperature range observed, E’ was higher in the gelatin 6 and 10 wt.% CNC composites, suggesting an increase in the strength in the composite material and confirming the reinforcement effect that was suggested with DSC results. A slight reduction in E’ at 50 °C could be related to the glass transition temperature associated with the amorphous fraction of the gelatin, as described in DSC. As mentioned in Section 1, results regarding cellulose mechanical reinforcement in gelatin matrices has been conflicting. The results obtained in this study confirms CNC mechanical reinforcement in a gelatin matrix by DSC and DMA, probably due to an increase in overall crystallinity on the composites determined by XRD; however, it is important to consider that different factors, such as cellulose origin and cellulose crystallinity, may be contributing to the composites’ mechanical properties. In this study, we used highly crystalline CNC from cotton, and not all the studies on cellulose reinforcement on a gelatin matrix analyze cellulose and overall composite crystallinity, making a direct comparison difficult. Also, the CNCs used in this study exhibited two different types of crystals (type I and II), and the effect of these on gelatin structure and mechanical reinforcement has not yet been elucidated. This is something that we aim to investigate in the future.

#### 2.2.7. Thermal Stability 

**Figure 8:**
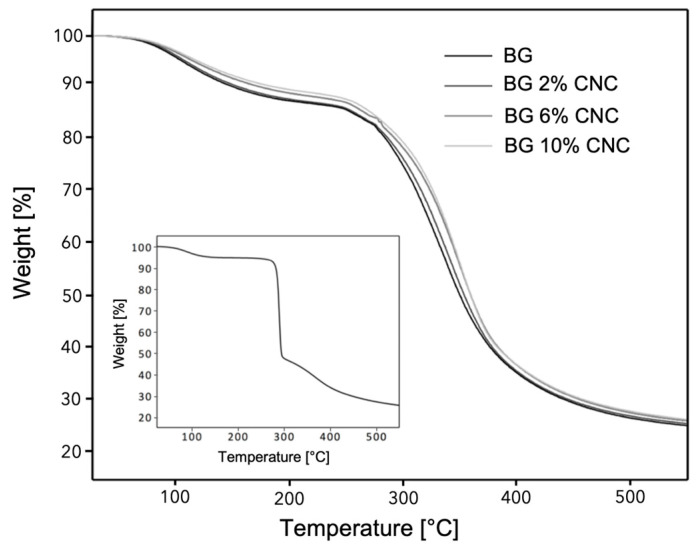
Thermograms of gelatin, CNC (inset), and gelatin–CNC composite films with various CNC weight percentages equilibrated at RH 33%.

TGA thermograms of the gelatin and gelatin–CNC composites showed three well-defined weight loss stages, where the first went up to approximately ~150 °C due to water evaporation, followed by a thermal degradation starting at ~250 °C (Figure 8). The third stage is related to the calcination of the films at ~400 °C. However, differences between the gelatin and composite films were observed. The onset and peak degradation temperatures increased with the addition of CNC, particularly in the case of BG-6 wt.% and 10 wt.% CNC composites (Table 5). This increase was higher than the value expected based on the weight fraction of CNC present in the composites and lower CNC peak degradation temperature (Figure 8, inset), indicating a clear synergism in thermal stability, possibly due to a molecular interaction between the two polymers. These results agree with previous results on gelatin–CNC, gelatin–CNF, gelatin–BC, and gelatin–MFC composites [32,42,43,44,74].

#### 2.2.8. Quantification of Shape Memory Properties

**Figure 9:**
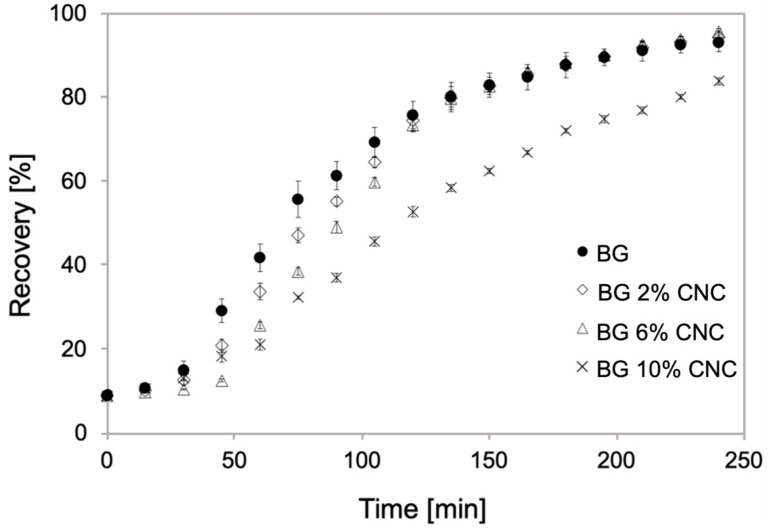
The percentage of the shape recovery of gelatin and gelatin–CNC composite films with different CNC weight fractions at 30 °C as a function of time.

To explore further the effect of gelatin and CNC interactions, we performed shape memory assays as described in the Section 4.3.8. If gelatin could improve its shape memory properties by the presence of CNC, it would suggest that the latter could act as a netting point promoter to improve fixing and shape recovery. In our samples, we observed in fact the opposite effect, where the presence of CNC decreased the kinetics and magnitude (in the case of 10 wt.% CNC) of the shape recovery (Figure 9). Since the improvement in shape recovery of the polymer can be related to its netting points, which are also related to molecular order [75], a decrease in crystallinity in the composite will then negatively affect its capacity of maintaining the original/permanent shape. Indeed, in our sample, CNC reduced the crystallinity of the gelatin, as indicated by XRD (Table 2), therefore affecting the recovery properties compared to the control (pure gelatin). Moreover, DSC showed a significant increase in Tg when the CNC was present, indicating a greater interaction of the polysaccharide nanoparticles with the amorphous fraction of the composite films.

Gelatin–CNC interactions has been established in our composites, showing that CNC presence (specifically 10 wt.%) is able to decrease water sorption as well as increase overall crystallinity of the composites as well as an increase in mechanical properties. Even though we carefully selected the conditions to prepare these composites, such as a controlled moisture content, constant pH, and low temperature (5 °C) (similar cooling rate expected among the sample), to promote both component interactions, these could still be improved to increase the range of their applications. One alternative for this improvement could be by using gelatin from other sources such as gelatin from cold-water fish. In fact, salmon gelatin has been shown to present lower melting and gelation temperatures than mammalian gelatin [76] as well as a higher molecular mobility than bovine gelatin [77]. Thus, this higher mobility could promote higher interactions when CNCs are present. In addition, pH and temperature are undoubtedly key factors for optimizing these interactions. The pH creates conditions under which both materials can combine and interact more effectively, given the strong negative charge commonly found in CNCs and the varying positive charge of gelatin, depending on its isoelectric point. Temperature influences the degree of triple helix formation during composite fabrication, promoting either a more amorphous or crystalline structure. By rationally combining these structures, we can optimize interactions and, as a result, better control the mechanical properties and structural stability of the composite. Indeed, it has been described that by changing the pH values while preparing the composites as well as the casting temperature could allow us to modulate gelatin–CNC physical properties and interactions [70]. Regarding CNCs, their surface modification can be a way to improve its interaction and compatibility with a polymer such as gelatin, with the aim of enhancing its shape memory properties. Some strategies for its modification include acetylation, oxidation, and silanization, which enhance their compatibility with various matrices [78]. Recent research has focused on CNC hybridization with materials like graphene, creating new composites with improved properties [79]. These approaches, or a combination of them, could help to develop composites with controlled properties that will depend on their desired application.

## 3. Conclusions

This work assessed the interactions between gelatin and CNC in composite films at low moisture content (~10 wt.% d.b, 33% RH) through molecular and thermomechanical characterizations. The CNCs exhibited high crystallinity, with type I and II crystal morphologies homogeneously distributed throughout the composites. FT-IR and XRD results demonstrated that the presence of CNCs interfered with the formation of the gelatin triple helix, reducing the specific crystallinity of the polymer. The overall crystallinity of the composites was found to increase due to the highly crystalline nature of CNCs. Water sorption studies showed that the monolayer in the composites decreased as overall crystallinity increased due to higher concentrations of CNC. This increase in crystallinity positively impacted the mechanical properties, with higher E’ values observed in DMA and improved thermal stability noted in TGA. The quantification of the shape memory ability of the gelatin and gelatin–CNC films indicated that while molecular interactions between the two components may occur, CNCs affected negatively the magnitude and kinetics of the shape recovery of the composites by reducing the netting point associated with the lower crystallinity of the gelatin, likely due to stronger interactions with the amorphous fraction of the polymer. We believe that the results presented in this study contribute to elucidating the interactions of gelatin–CNC composites at various structural levels and highlights that even though CNCs act as reinforcement materials on gelatin matrices, their interactions are complex and do not imply synergism in their properties, and these properties can depend on pH, temperature, and moisture content as previously discussed. Further, investigation is, however, needed to fully understand CNC–gelatin interfacial interactions, particularly the effects of CNC’s weight fraction and crystal polymorph on the resulting mechanical properties. This knowledge is crucial for the development of advanced biomaterials for food and packaging applications, where the use of natural and edible materials presents a clear advantage.

## 4. Materials and Methods

### 4.1. Materials and Reagents

Bovine gelatin (bloom 200) was purchased from Rousselot (Sao Paulo, Brazil). Cellulose nanocrystals (CNC) (dimensions 5–20 nm wide, 150–200 nm long) from cotton cellulose pulp were purchased from University of Maine, Process Development Center (Orono, ME, USA). Phosphorous pentoxide (P_2_O_5_), potassium chloride (KCl), and magnesium chloride (MgCl_2_) were purchased from Merck (Darmstadt, Germany).

### 4.2. Gelatin–CNC Composite Preparation

The composites’ preparation was adapted from the methodology described by Quero et al., 2015 [32]. First, 7% *w*/*v* aqueous gelatin suspensions were prepared by dissolving 25.2 g of gelatin into 360 mL of distilled water. The suspensions were stirred with a magnetic bar for 1 h at 55–60 °C to assure the complete dissolution of the gelatin powder. Simultaneously, a CNC 0.01% *w*/*v* aqueous suspension was prepared by stirring for 30 min at room temperature and sonicated for 3 min at 25 °C at a frequency of 40 kHz (Isolab, Eschau, Germany) to promote CNC dispersion. Different volumes of the CNC suspension were added to the gelatin suspensions to achieve 2, 6, or 10 wt.% of CNC with respect to dry gelatin. These concentrations were chosen based on our previous study using gelatin–bacterial cellulose composites [32]. The gelatin–CNC suspensions were stirred with a magnetic bar for 1 h. Finally, pure gelatin (control) and gelatin–CNC suspensions were casted onto 90 mm diameter polystyrene Petri dishes and subsequently stored for 2 weeks at 5 °C to allow for water evaporation and obtain films with a final thickness of 0.20 ± 0.03 mm. After casting, the films were further dried in a desiccator containing P_2_O_5_ until use. For molecular interactions, surface mapping, thermal, and mechanical analysis, samples were then equilibrated under a MgCl_2_ saturated solution (33% RH) for 3 to 4 weeks to obtain films with defined moisture content (~10 wt.% d.b). It is important to note that these composites still contain enough water to generate some structural mobility and avoid brittleness while maintaining their structural integrity during handling. Casting and further moisture equilibrations of the prepared composites were carried out in the presence of thymol to prevent fungal growth. The moisture content of all films was determined gravimetrically in triplicate by drying at 105 °C for 24 h.

### 4.3. Characterization of Gelatin–CNC Composites

#### 4.3.1. Molecular Interactions by Fourier Transform Infrared Spectroscopy (FT-IR)

The presence of CNC in the composite films and the interaction between components was assessed by FTIR spectroscopy (Tensor-27, Bruker Optics Inc., Billerica, MA, USA) equipped with diamond attenuated total reflectance (ATR). The spectra were obtained over a range of 4000–500 cm^−1^. Each spectrum was obtained using 128 scans, using 4 individual samples for each composite. CNC powder was analyzed using the same experimental conditions.

#### 4.3.2. Surface Mapping by RAMAN Spectroscopy

Surface mapping of gelatin–CNC composite films were analyzed with a Raman spectrometer (XploRA PLUS, Horiba Scientific, Palaiseau, France). The Raman spectrometer was equipped with a near infrared laser operating at a wavelength of 785 nm with a beam diameter of 1 μm. The laser power used was 70 mW, which did not induce sample burning. Gelatin and CNC spectra were acquired using a diffraction grating with a groove density of 600 gmm^−1^. The laser was focused on the sample’s surface using an optical microscope (Olympus BX41, Eugene, OR, USA) with a ×50 long-working distance objective (PL Fluotar, NA = 0.55). Each spectrum was acquired in the wavenumber range of 200–2000 cm^−1^ using an exposure time of 30 s and 1 cycle. Raman shifts were calibrated with the silicon reference peak at 520.7 cm^−1^. All spectra were corrected using the instrument software (LabSpec 6 software version 6.4, Horiba Scientific, Kyoto, Japan). The Classical Least Squares (CLS) fitting procedure was used to create an image based on the component distribution.

#### 4.3.3. Crystallinity by X-Ray Diffraction (XRD)

The crystallinity of gelatin, CNC, and gelatin–CNC composite films were obtained using an X-ray diffractometer (Phillips X’Pert Pro, Nashville, TN, USA) with a CuKα radiation source (1.541 A°). Films were not minced to avoid possible changes in the films microstructure. The spectra were recorded from 2θ = 5 to 40° using a step size of 0.02°, a current of 30 mA, and a voltage of 40 kV. All diffractograms were normalized using the software OriginPro8 SR0 V8.0724 (BT24, Northampton, MA, USA). The crystallinity index (Χc) of CNC was determined using the integration method and the equation [80].

(1) $$\chi_{C}=\frac{A_{C}}{A_{C}+A_{A}}\times 100,$$

where A_C_ and A_A_ are the areas under the X-ray diffraction pattern that correspond to the contribution of crystalline and amorphous regions, respectively [32,80]. Regarding the XRD patterns of pure gelatin and gelatin–CNC composites, the area under the peaks located at 2θ~7° (crystalline fraction) and 2θ~15–25° (amorphous fraction) were calculated by integration using the software OriginPro8 SR0 V8.0724 (BT24). Then, the crystallinity of gelatin and gelatin–CNC composites was calculated using Equation (1).

#### 4.3.4. Water Interactions by Dynamic Vapor Sorption (DVS)

Water sorption of the composite films at various relative humidities was determined using a Dynamic Vapour Sorption system (DVS-INTRINSIC, Surface Measurement Systems Ltd., Wembley, UK). The method used was adapted from [72]. Pure gelatin and the gelatin–CNC composite films were stored over P_2_O_5_ for 2 weeks and then cut into small pieces (~1.5 × 1.5 mm). Approximately 10 mg of each sample were used for each measurement. Samples were dried under nitrogen flow at 20 °C and then equilibrated from 0% to 90% relative humidity (HR) at 10% increment RH steps. The equilibrium criteria at each step was defined when dm/dt (change in mass% with time) = 0.002% min^−1^. After the measurement, the sorption isotherms (expressed on a dry basis) were numerically fitted using the Guggenheim, Anderson, and de Boer (GAB) equation [73]:

(2) $$M=\frac{m_{o}C_{GAB}Ka_{w}}{\left(1-Ka_{w})(1-Ka_{w}+C_{GAB}Ka_{w}\right)},$$

where M is water content on a dry basis, m_o_ corresponds to the moisture content at the monolayer, C_GAB_ corresponds to the constant associated to the heat of sorption of water molecules interaction at the monolayer, K is the constant associated to the heat of sorption of water at the multilayer, and a_w_ is the water activity or RH at thermodynamic equilibrium [81,82]. The fitting of the GAB equation was achieved by minimization of the sum of the square differences between the experimental and predicted values using the Solver Excel tool (Office 2016; Microsoft Corp., Redmond, WA, USA). An adequate fitting of the data was confirmed by calculating the mean standard error (MSE):

(3) $$MSE=\frac{100}{N}\sum_{i=1}^{N}\left|\frac{m_{ei}-m_{pi}}{m_{ei}}\right|,$$

where m_ei_ corresponds to the experimental value, m_pi_ is the predicted value, and N denotes the number of experimental points [83,84].

#### 4.3.5. Thermal Stability by Thermogravimetric Analysis (TGA)

Thermal stability of gelatin, CNC, and gelatin–CNC composites were analyzed using TGA/DSC3^+^ (Mettler Toledo, Greifensee, Switzerland). Briefly, ~10 mg of each sample was weighed into 100 µL aluminium crucibles with pierceable lids. Before the experiment, the pan lids were automatically pierced to allow for escape of vapors, and the samples were heated from 20 to 550 °C at a rate of 10 °C/min in a N_2_ atmosphere, with a N_2_ flow rate of 25 mL/min. Onset degradation temperature was determined by extrapolation, and the peak degradation temperature was determined from the first derivative of the weight as a function of change in temperature using STARe Software (SW V15.00).

#### 4.3.6. Thermal Characterization by Differential Scanning Calorimetry (DSC)

The thermal properties of the composites were assessed by differential scanning calorimetry (DSC 1 STAR System, Mettler-Toledo, Switzerland) using an intracooler TC100 (HUBER, Offenburg, Germany). The instrument was calibrated using indium (Tm = 156.6 C and ΔH = 28.55 J/g). Approximately 20 mg of each sample was weighed into an aluminium crucible (40 µL) and then hermetically sealed. The experimental protocol used was as follows: cooling from 25 °C to −50 °C at 40 °C/min, isothermal step at −50 °C for 15 min, and heating to 110 °C at 10 °C/min, followed by an isothermal step at 110 °C for 3 min. An empty pan was used as a reference, and N_2_ was used as a purge gas. The glass transition (T_g_) and melting (T_m_) temperatures and changes in the melting enthalpy (∆H_m_) were determined using STARe Software (DB V12.10) considering T_g_ and T_m_ as the midpoint and onset temperatures, respectively.

#### 4.3.7. Mechanical Characterization by Dynamic Mechanic Analysis (DMA)

The mechanical properties of the different composite films under dynamic conditions were determined using a DMA-1 Instrument (Mettler-Toledo, Greifensee, Switzerland). Composite films were cut into strips (~7 × 20 mm) and covered with high vacuum grease (Dow Corning, Midland, Michigan, USA) to avoid moisture loss during analysis. The instrument was set in tension mode, and measurements were obtained within the linear viscoelastic region of the sample. A temperature scan from 5 °C to 90 °C at a heating rate of 3 °C/min and a frequency of 10 Hz were used. The parameters recorded were the elastic modulus (E’). At least five replicates were measured for each sample.

#### 4.3.8. Shape Memory Assays

Films were cut into 1 × 7 cm rectangular strips, and the shape memory tests were carried out by bending and fixing the samples at 40% RH using a KCl saturated solution, and they were stabilized for one week at 30 °C. Then, the RH was reduced to approximately 7% using silica gel and stabilized for another week at 30 °C. Finally, the composites were loosened, and RH was increased again to 40%. The recovery angle of the samples was measured during a maximum period of 4 h at 30 °C, with photographs being taken every 15 min until no changes were observed for half an hour in the images. Images obtained were analyzed using ImageJ to determine the recovery angles. The percentage of shape was calculated as described [85] using the following formula:

(4) $$R\%=\frac{\theta i-\theta f}{\theta i}\cdot 100$$

where R%: percent of recovery, θi: initial angle after bending and fixation, and θf: final angle after removing fixation.

### 4.4. Statistical Analysis

When pertinent, statistical analysis was assessed using one-way ANOVA with Tukey multiple comparisons using the software GraphPad Prism 9.5.1 (CA, USA). *P*-values less than 0.05 were considered to be statistically significant. * *p* < 0.05 ** *p* < 0.005, *** *p* < 0.0005.

## Figures and Tables

**Table 1:** The identification of the amide A and amide I absorption peaks by FT-IR in gelatin and gelatin–CNC composite films with various CNC weight percentages.

Assignation	Wavenumber Position (cm^−1^)			
	BG	BG 2%CNC	BG 6%CNC	BG 10%CNC
Amide A	3289	3291	3292	3291
Amide I	1627	1629	1628	1628

**Table 2:** Normalized peak areas and percentage of crystallinity for gelatin and gelatin–CNC composite films determined by X-ray diffraction.

Sample	Normalized Peak Area at 2θ = 7° (a.u/°)	Gelatin Crystallinity (%)	Composite Crystallinity (%)
BG	1.86	21.73	21.73
BG 2%CNC	1.55	18.11	21.41
BG 6%CNC	1.59	18.57	25.24
BG 10%CNC	1.03	12.03	22.58

**Table 3:** GAB fitting parameters of sorption isotherms at 20 °C of pure gelatin, pure CNC, and gelatin–CNC composite films with different CNC weight percentages.

Sample	m_o_ (%)	C_GAB_	K	MRE (%)
BG	10.50	6.99	0.81	2.71
BG 2%CNC	10.18	9.38	0.82	1.06
BG 6%CNC	9.61	10.23	0.83	1.20
BG 10%CNC	9.52	9.71	0.82	0.98
CNC	4.38	9.95	0.91	2.36

**Table 4:** Glass transition temperature (Tg), melting temperature (Tm), and change in enthalpy of melting (ΔHm) for gelatin and gelatin–CNC composite equilibrated at RH 33%.

Sample	Tg (°C)	Tm (°C)	ΔHm (Jg^−1^)
BG	45.15 ± 0.96	89.36 ± 0.24	27.98 ± 0.33
BG 2%CNC	48.64 ± 0.41	94.19 ± 2.01	29.20 ± 1.26
BG 6%CNC	48.64 ± 0.31	94.39 ± 0.86	29.25 ± 0.23
BG 10%CNC	48.46 ± 1.57	94.64 ± 1.03	28.88 ± 0.83

**Table 5:** The degradation onset temperature and degradation peak temperature from TGA measurements determined from the first derivative of the weight as a function of the change in temperature. Different letters show significant differences between samples (*p* < 0.05).

Sample	Moisture Content (%)	Onset Temperature (°C)	Peak Temperature (°C)
BG	12.6 ± 0.7	285.7 ± 9.0 ^a^	326.4 ± 12.4 ^a^
BG 2%CNC	13.1 ± 0.2	290.0 ± 5.5 ^a^	339.4 ± 5.2 ^a,b^
BG 6%CNC	11.9 ± 0.2	299.5 ± 0.5 ^a^	352.0 ± 0.3 ^b^
BG 10%CNC	12.2 ± 0.7	302.7 ± 0.9 ^a^	352.6 ± 1.4 ^b^
CNC	7.3 ± 0.5	286.3 ± 0.2 ^a^	291.7 ± 0.4 ^c^

## Data Availability

All data and materials are available on request from the corresponding author. The data are not publicly available due to ongoing researches using a part of the data.
